# Diagnostic and Therapeutic Value of ERCP in Acute Cholangitis

**DOI:** 10.1155/2013/191729

**Published:** 2013-08-13

**Authors:** Kenan Buyukasik, Ahmet Burak Toros, Hasan Bektas, Aziz Ari, Mehmet Mehdi Deniz

**Affiliations:** ^1^Department of General Surgery, Istanbul Education and Research Hospital, 34098 Istanbul, Turkey; ^2^Department of Gastroenterohepatology, Istanbul Education and Research Hospital, 34098 Istanbul, Turkey

## Abstract

Cholangitis, with a clinical spectrum between acute ascending cholangitis and acute fulminant cholangitis, the mildest and the most severe forms, respectively, is the infection of bile ducts with a potential of serious mortality and morbidity. Obstruction of the bile ducts followed by infection, with *E. coli* being the most commonly isolated agent, is common to all forms of cholangitis. Biliary obstruction is caused by choledocholithiasis mostly. “Choledochal pressure” is the most important factor, determining morbidity. If the pressure exceeds 25 cm H_2_O, which is the critical value, immune dysfunction ensues. Sepsis is common if the infection of biliary ducts is suppurative. Mortality and morbidity are inevitable if left untreated or drained late. The objective of this study is, in the stand point of the current literature, to analyse the diagnostic, therapeutic success and complication rates of ERCP (Endoscopic retrograde cholangiopancreatography) in patients with a diagnosis of acute purulent cholangitis with no response to medical treatment.

## 1. Introduction

Inflammation of the biliary ducts is called cholangitis. Inflammatory process usually begins extrahepatically and easily spreads intrahepatically, causing bacteriemia. Cholangitis was first defined by Charcot in 1877. Obstruction of the biliary ducts and presence of a superposing bacterial infection are common features in cholangitis. The mildest clinical form is ascending cholangitis, and the most severe form is acute fulminant cholangitis. Not every biliary obstruction is associated with cholangitis but there is surely a biliary obstruction in every cholangitis case [1–4].

“Reynolds pentad” was defined with the addition of mental confusion and septic shock, in 1959, by Reynolds and Dragon, to the clinical findings known as “Charcot triad” (fever, abdominal pain, and jaundice) [5, 6].

Biliary obstruction is caused by choledocholithiasis mostly. Moreover, malignancy, benign strictures, and interventions to the biliary ducts may be the cause of biliary obstruction. Bacterial contamination of the biliary ducts may be caused by ascending infection or portal bacteriemia. That means cholangitis clinically. “Choledochal pressure” is the most important factor, determining morbidity. If the pressure exceeds 25 cm H_2_O, which is the critical value, hepatic defence mechanisms against infection are disrupted and immune dysfunction ensues.

Obstruction of the biliary ducts, increased intraluminal pressure, and infected bile are important in the pathogenesis of cholangitis. In 25–40% of the cases, associated with the presence of choledochal pressures exceeding 25 cm H_2_O infection spreads into the intrahepatic canalicules, and cholangiovenous reflux ensues, followed by the access to the hepatic veins and lymphatics, resulting in bacteriemia. Sepsis is common in the case of suppurative infections [7]. This clinical picture, which used to have a 80–90% mortality in the past, has serious morbidity without treatment or with delayed treatment, and the mortality rate has decreased to only 5–15% despite interventions using endoscopic retrograde cholangiopancreatography (ERCP) and potent antibiotics. 

Mortality rate is reported to be between 17% and 40% in the presence of accompanying medical problems and advanced age. Mortality is decreased significantly with endoscopic drainage, performed after stabilization of the patients [5, 8]. 

The objective of this study is, in the light of current literature, to analyse the diagnostic, therapeutic success and complication rates of ERCP in patients with a diagnosis of acute purulent cholangitis, without response to medical treatment.

## 2. Material and Method

50 patients who had been diagnosed with acute suppurative cholangitis and underwent ERCP, between years 2010 and 2011 in Istanbul Education and Research Hospital, were reviewed. 24 of the patients were males and 26 were females, with a median age of 58 (28–83). 

Tokyo criteria were used for the diagnosis of cholangitis. All the patients underwent biochemical and microbiologic tests, before and after the intervention. Tumor markers were also demanded for suspect cases and the ones with advanced age. In the presence of an indication for transabdominal USG, all the patients underwent CT or MRCP.

Tokyo criteria were also used for assessment of the severity and planning of the treatment [8] (Algorithm 1). Based on the Tokyo criteria, patients with a certain diagnosis or who have a deteriorating general condition despite intense supportive treatment underwent biliary drainage with ERCP. 

Procedures were performed using a Fujinon ED 450x4 duodenoscope (side viewing) and a Siemens Siremobil Compact L radiology tool. Prophylactic antibiotic was administered to all the patients, before the procedure. Sedation was applied with the appropriate doses of midazolam and meperidine. Also, appropriate doses of N-butyl-bromide were administered when needed. Standard equipment was used during the ERCP procedures. 10 cm biliary stents of 8.5 or 10 F diameter was used for the patients in need.

Pre- and post-interventional clinical and laboratory findings, diagnostic and therapeutic success of ERCP, and complications of the procedure were studied in the light of the current literature.

**Algorithm 1**

Tokyo criteria in diagnosing cholangitis:

- *Clinical Findings.* History of bile duct disease, fever, chills, jaundice, and abdominal pain (especially at right upper quadrant).
- *Laboratory Findings.* Signs of inflammatory response (increasing leukocyte count, elevated CRP) abnormal liver function tests (increased ALT, AST, ALP, and GGT).
- *Imaging Studies.* Dilated bile ducts or etiologic proof (stricture, stone, stent, etc.).
  - *Suspect Diagnosis*: 2 or more findings from group A.
  - *Certain Diagnosis*: 2 or more findings from group A and findings from groups B and C.

## 3. Results

Basic factors in the pathogenesis of acute cholangitis are obstruction of the biliary ducts causing increased intraluminal pressure and eventually infection of the bile. Obstruction of the bile ducts is surely present in every cholangitis case. In our study, diseases causing obstruction of the bile ducts at the patients with a diagnosis of purulent cholangitis based on Tokyo criteria are shown in Table 2.

60 ERCP procedures were performed in 50 patients. Success rate of the endoscopic treatment in 50 patients was 94% (47) (Table 1). Open surgery was performed in 2 patients with multiple stones larger than 2 cm; percutaneous drainage was performed in 1 patient with a diagnosis of cholangiocarcinoma, due to failed cannulation (Table 3). No complications or mortality was seen due to the procedure. 

There were no problems in the follow-up controls (3 months after the stents were removed) of the 17 patients, who underwent stenting. Elective cholecystectomy was performed in 36 patients with cholelithiasis. Metallic biliary stent was applied to 3 of the 4 patients with cholangiocarcinoma, who were decided as inoperable. 

Clinical findings and laboratory parameters were significantly relieved, following the endoscopic interventional procedures (Table 4).

ERCP is an important method for visualising the biliary ducts, based on its diagnostic and therapeutic features. Success rates of this method were reported to be 98% and were found to be 94% in our study. In this method, which is safer than surgical and percutaneous interventions, the complication rate is reported to be 1.38%, with mortality rate being 0.21%. There were no complications in our study group.

## 4. Discussion

Cholangitis, with a clinical range between acute ascending cholangitis and acute fulminant cholangitis, the mildest and the most severe forms, respectively, is the infection of bile ducts with serious mortality and morbidity. Obstruction of the bile ducts followed by infection is common to all forms of cholangitis. Isolated organisms are *E. coli* (27%), *Klebsiella* (16%), *Enterococcus* (15%), *Streptococcus* (8%), *Enterobacter* (7%), and* Pseudomonas aeruginosa* (7%) [3, 4, 9, 10].

Although interventional procedures such as ERCP decrease the mortality and morbidity rates there are opinions stating that these procedures being used increasingly in the recent years may themselves cause bile contamination hence the incidence of cholangitis [4, 11, 12].

Drainage should not be delayed in patients with advanced age and accompanying medical problems. If delayed, mortality is reported to be between 17%–40%. The mortality rate is reduced to 3% with an elective drainage procedure, performed after the stabilization of the patient. In our series, Tokyo criteria were used for assessment of the level of disease and the timing of treatment. According to Tokyo criteria, patients with 2 or more findings from group A and with findings from groups B and C were diagnosed with cholangitis and underwent endoscopic drainage.

Normally, pressure of the hepatic bile secretion is 120–150 cm H_2_O and the pressure in extrahepatic bile ducts is 100–150 cm H_2_O. Normally, bile secretion occurs according to these pressure values and bile fills into the gallbladder with a presssure of 12–18 cm H_2_O. Peristaltic contraction relaxation of the sphincter of Oddi is the most important factor in the regulation of this pressure. Bile secretion from liver is inhibited if the pressure exceeds 300 cm H_2_O. If the choledochal pressure exceeds 25 cm H_2_O, hepatic defence mechanisms against infection become useless.

Cholangitis may cause clinical pictures changing from mild forms to severe fulminant forms, that may be followed by sepsis [4–7, 13]. It is reported that diagnosis may be missed in up to 25% of the patients, presenting with sepsis [4, 13]. Cholangitis must be kept in mind especially in septic, elderly patients with abdominal pain and jaundice.

Clinical findings found in the literature are fever in 90% of the cases but may be absent in elderly patients. Fever was present in 80% of our patients. Abdominal pain, especially in the right upper quadrant, was found in 80% of the patients; the rate was 78% in our series. Jaundice was reported to be 60% in the literature but was found to be 90% in our series. This difference is attributed to the fact that our hospital is a reference center. Hypotension and mental disorders were reported to be between 15% and 30% in the literature; in our study the rate was 25%–35%.

Laboratory findings in acute cholangitis reveal findings of inflammation and biliary stasis. In 80% of cholangitis cases, leukocytosis was reported to be >10.000/mm in our series the average leukocyte count was found to be 9.600/mL before the procedure. ALP was found to be increased in 70% of cases, which was concordant with the 78% rate reported in the literature. Bilirubin levels were high in almost all cases. 

CRP and sedimentation rate were typically increased. Calcium levels must be evaluated for possible pancreatitis, as well as electrolyte levels for renal functions. Coagulation tests (high in cirrhosis and DIC) for patients with possible need for interventions and tumor markers for patients with advanced age must also be evaluated. 

Transabdominal USG was performed in all patients, also CT and/or MRCP were performed as needed. USG revealed biliary stone in the choledochus of 15 patients (30%). In a study on cholangitis and USG, although the dilatation of choledochus was seen in 70%, biliary stone was reported to be seen only in 13%. Therefore, the presence of cholangitis can not be excluded by USG only. 

MRCP is a noninvasive diagnostic method, being used increasingly for investigation of gallstones and pathologies of the biliary ducts. But, its sensitivity is low for stones less than 6 mm in diameter. In our series, biliary duct pathology was revealed in 15 (83%) of 18 patients, who underwent MRCP. 

14 patients underwent CT. Its success for revealing stones in the biliary ducts was 42%. Despite this low success rate, the advantage of CT relies on its ability to display the causes and complications of cholangitis.

## 5. Conclusions

Emergency bile drainage must be performed in patients without response to medical treatment, with findings of sepsis and jaundice, with possible obstruction and cholangitis. In our study, the effectiveness of endoscopic treatment for biliary drainage was confirmed. After endoscopic treatment, clinical and laboratory findings were relieved dramatically.

In conclusion, in acute purulent cholangitis, timely performed endoscopic interventions (ERCP) are still a reliable option with increased diagnostic and therapeutic effectiveness and decreased morbidity and mortality rates, as compared to other methods for the evaluation of biliary ducts.

## Figures and Tables

**Table 1:** Clinical findings. *N*: 50.

	Before procedure: *n* (%)	After procedure: *n* (%)
Jaundice	45 (90%)	3 (6%)
Pain (especially at right upper quadrant)	39 (78%)	4 (8%)
Fever	40 (80%)	10 (20%)
Hypotension, disturbed consciousness	12 (24%)	0

**Table 2:** Primary diseases found in cases.

	*n* (%)
Choledocholithiasis	40 (80%)
Cholangiocarcinoma	4 (8%)
Cyst hydatid	4 (8%)
Biliary stricture (due to previous laparoscopic cholecystectomy)	1 (0.5%)
Long standing stent (placed at the previous ERCP session)	1 (0.5%)

**Table 3:** Endoscopic procedures performed. *N*: 50 (more than one method is used in some cases).

	*n* (%)
Endoscopic sphincterotomy, lithotripsy, and stone extraction with balloon or basket catheter.	41 (82%)
Stenting	17 (34%)
Precut sphincterotomy	4 (8%)
Nasobiliary drainage	2 (4%)
Failed intervention	1 (1%)
Biliary stone that could not be extracted endoscopically (referred for surgery)	2 (4%)

**Table 4:** Laboratory parameters of patients before the procedure and 72 hours after the procedure.

Laboratory values	Before procedure	72 hours after procedure
Total bilirubin	9.5 (1.8–18.6)	2.4 (1.4–5.8)
Direct bilirubin	4.8 (1.4–9.6)	1.3 (0.8–2.3)
ALT (U/L)	55 (16–285)	45 (12–143)
Leukocyte (cell/mm^3^)	9.600 (4.500–23.000)	6.400 (4.200–12.000)
